# Ongoing outbreak of invasive and non-invasive disease due to group A *Streptococcus* (GAS) type *emm*66 among homeless and people who inject drugs in England and Wales, January to December 2016

**DOI:** 10.2807/1560-7917.ES.2017.22.3.30446

**Published:** 2017-01-19

**Authors:** Nick Bundle, Laura Bubba, Juliana Coelho, Rachel Kwiatkowska, Rachel Cloke, Sarah King, Jill Rajan-Iyer, Max Courtney-Pillinger, Charles R Beck, Vivian Hope, Theresa Lamagni, Colin S Brown, Daiga Jermacane, Rachel Glass, Monica Desai, Maya Gobin, Sooria Balasegaram, Charlotte Anderson

**Affiliations:** 1 United Kingdom Field Epidemiology Training Programme, Public Health England, United Kingdom; 2 Field Epidemiology Services, National Infection Service, Public Health England, London and Bristol, United Kingdom; 3 European Programme for Intervention Epidemiology Training (EPIET), European Centre for Disease Prevention and Control (ECDC), Stockholm, Sweden; 4 Reference Department Microbiology Services Division, National Infection Service, Public Health England, London, United Kingdom; 5 European Programme for Public Health Microbiology Training (EUPHEM), European Centre for Disease Prevention and Control (ECDC), Stockholm, Sweden; 6 Respiratory and Vaccine Preventable Bacteria Reference Unit, National Infection Service, Public Health England, London, United Kingdom; 7 United Kingdom Public Health Specialty Training Programme; 8 Public Health England South East, Horsham, United Kingdom; 9 Public Health England South West, Bristol, United Kingdom; 10 Public Health Institute, Liverpool John Moores University, Liverpool, United Kingdom; 11 Healthcare-Associated Infection & Antimicrobial Resistance Department, National Infection Service, Public Health England, London, United Kingdom; 12 HIV and STI Department, National Infection Service, Public Health England, London, United Kingdom

**Keywords:** invasive streptococcal infections, people who inject drugs, epidemiology, outbreak, homelessness, Emm66

## Abstract

We report an outbreak of invasive and non-invasive disease due to an unusual type of *Streptococcus pyogenes(*group A *Streptococcus*, *emm*66) among a vulnerable, largely homeless population in southern England and Wales, detected in September 2016. Twenty-seven confirmed cases were subsequently identified between 5 January and 29 December 2016; 20 injected drugs and six reported problematic alcohol use. To date, we have ruled out drug-related vehicles of infection and identified few common risk factors.

On 26 September 2016, a cluster of invasive disease caused by *Streptococcus pyogenes* (group A *Streptococcus,* GAS) was detected among people who inject drugs (PWID) or who were street homeless in a town in the south of England. A local outbreak control team (OCT) was set up to investigate this cluster which included infections due to *emm*66, a GAS type rarely identified by the Public Health England (PHE) Respiratory and Vaccine Preventable Bacteria Reference Unit (RVPBRU) in previous years. Additional cases of both invasive (iGAS) and non-invasive disease due to GAS type *emm*66 were retrospectively identified in the RVPBRU database and a review of local health protection team (HPT) case records revealed the majority of them to have occurred among PWID, those homeless, or reporting problematic alcohol use. A national OCT was convened on 14 October 2016 with representation from local and national health protection, epidemiology and microbiology services. We describe the ongoing outbreak of invasive and non-invasive disease caused by GAS *emm*66 as at 12 January 2017.

## Epidemiological investigation and microbiological characterisation

The outbreak case definition is individuals with confirmed GAS type *emm*66 infection (invasive and non-invasive) in England and Wales with a sample date from January 2016, who are, or are epidemiologically linked to someone who is, homeless, PWID or reporting problematic alcohol use.

Cases were identified from notifications made to HPTs in England and Wales [1,2] and from typed isolates from RVPBRU. Invasive disease was defined through the isolation of GAS from normally sterile sites.

We gathered information on lifestyle risk factors, including alcohol use and vehicles of infection related to drug use or homelessness, potential venues and modes of transmission, from a hypothesis-generating questionnaire. Questions covered accommodation, social contacts and drug use, including injection practices, in the seven days before illness and in the past year. We used validated questions, where possible, from the United Kingdom (UK)’s Unlinked Anonymised Monitoring Survey (UAM) of PWID [3] so as to provide comparator data. Questionnaires were completed by local HPTs and homeless/drug outreach services. We also summarised information obtained from outreach services, case records, local investigations and laboratory surveillance.

Initial testing for GAS usually occurs at local hospital laboratories that forward most isolates from invasive infections to RVPBRU for further characterisation. In this outbreak, laboratories were also encouraged to forward isolates from non-invasive infections in people who might meet the case definition. GAS types were determined by the *emm* sequence typing method [4], which compares the *emm* sequences obtained by PCR and Sanger sequencing to those available in the *emm* database using the BLAST algorithm [5].

## Description of the outbreak

**Figure 1:**
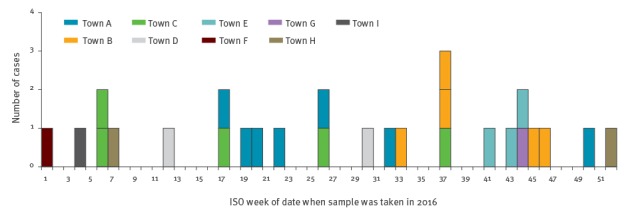
Outbreak cases of group A *Streptococcus* type *emm*66 infection, by week of sample date and town of residence, England and Wales, 2016 (n=27)

RVPBRU identified 30 *emm*66 infections in 2016, of which 27 (iGAS: 20; non-invasive GAS: 7) met the outbreak case definition with samples taken between 5 January and 29 December 2016 (Figure 1).

ISO: International Organization for Standardization.

As at 12 January 2017, 12 questionnaires were returned; ten fully completed from case interviews and two partially completed from case information and interview with a homeless outreach worker. Demographic and clinical characteristics of cases are summarised in Table 1.

**Table 1:** Features of outbreak group A *Streptococcus* type *emm*66 cases, England and Wales, 5 January–29 December 2016 (n = 27)

Total cases	n
**Demographics and risk factors^a^**	
Male	22
Median age (range) in years	38 (29–56)
Homeless at time of illness onset	20
Street homeless at time of illness onset	13
People who inject drugs	20
Problematic alcohol use	6
**Initial clinical presentation**	
Abscess	7
Injection site infection	6
Septic arthritis	3
Bacteraemia	3
Muscle or deep tissue infection	3
Cellulitis	2
Unspecified soft tissue infection	1
Necrotising fasciitis	1
Pneumonia	1
**Diagnosis, outcomes and co-infection^a^**	
Invasive GAS infection	20
Non-invasive GAS infection (including three severe infections)	7
Hospitalisation	21
Amputation	1
Died due to GAS infection	1
Previously tested positive for hepatitis C	15
Previously tested positive for hepatitis B	1

GAS: group A *Streptococcu*s.

^a^ More than one answer could be chosen.

**Figure 2:**
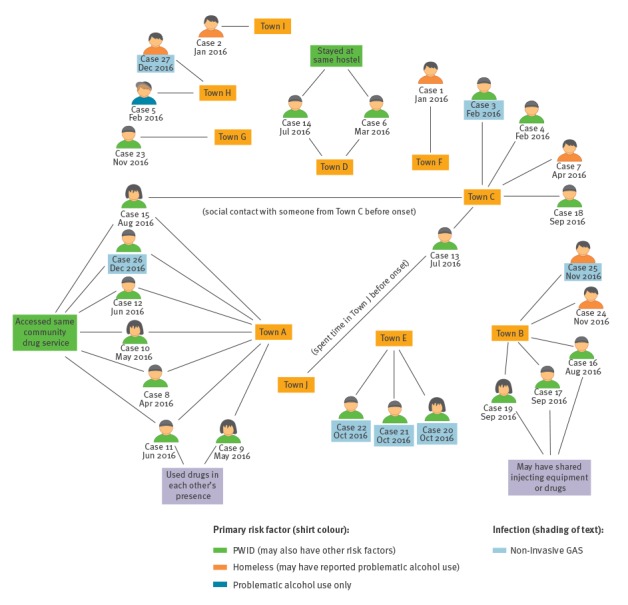
Network map summarising links between cases by town, place and social contact, outbreak of group A *Streptococcus* type *emm66*, England and Wales, 5 January–29 December 2016 (n = 27)

GAS: group A *Streptococcus*; PWID: people who inject drugs.

The cases were predominantly clustered across a 280 km span of southern England and Wales, bounded by Towns B and C to the east and Town H to the west. Nine towns in total had cases. Towns A, C, D, E, F, G and H are located along, or near to, a major road and rail transport corridor that connects London and South Wales. Town B is linked to this corridor via Town C. Town I is situated in the north of England. (Figure 2).

In Town A, all seven cases were linked through drug use or access to the same community drug service and six of these cases were also clustered in time (April–August 2016). All cases in Town B were street homeless, with three cases linked in time (August–September) and through anecdotal reports of sharing needles or drugs. All cases in Town C appeared unconnected. A common homeless hostel linked cases in Town D and information is pending about Town E. There is no known staff travel between hostels in different towns. There was social contact by one case between Towns A and C whose onset was among the latest in each of these locations. Another Town C case reported spending time, before onset of illness, in Town J, located 40 km from Town A.

Among the ten cases interviewed, the median delay between the sample and interview date was 104.5 days (range 3–263). Six of these interviewees were retrospectively identified cases and a much shorter median delay of 8.5 days (range 3–27) was seen among the four prospectively identified cases. (Table 2).

**Table 2:** Summary of responses from interviews with group A *Streptococcus* type *emm*66 outbreak cases, England and Wales, 2016 (n = 10)

Positive responses	n
**Country of origin, accommodation and contacts in the seven days before illness onset^a^**	
Born in the UK	9
Stayed in a house or flat (own, partner's or friend's)	6
Stayed outside (street, park or abandoned building)	5
Stayed in a squat	3
Stayed in a hostel	1
Accessed community drug or health services	6
Knew someone suffering from the same illness, infection or sore throat at a similar time	5
Visited or spent time with someone from another part of the UK	3
**Drug use and behaviour in the seven days before illness onset^a^**	
**Any drug use**	10
**Injecting drug use^a^**	8
Heroin	8
Crack	6
Used spoons/mixing containers or filters previously used by someone else	4
Changed drug dealer	2
**Other drug use^a^**	8
Smoked crack	7
Smoked, chased or snorted heroin	6
Smoked or swallowed cannabis	3
Snorted cocaine	2
Swallowed non-prescribed benzodiazepines	2

UK: United Kingdom.

^a^ More than one answer could be chosen.

All ten cases reported drug use in the seven days before illness, with the eight PWIDs mainly using heroin and crack. Some reported sharing of spoons/mixing containers and filters, but not needles. Two PWIDs linked to different towns (A and D) reported having changed dealers in the seven days before illness onset. Otherwise, there was no notable change in reported injecting behaviour or other drug use compared with that during the year prior to questionnaire administration.

## Control measures

We cascaded a health alert on 2 November 2016 to HPTs, microbiology services and local authorities working with affected populations to highlight the need for early detection of infection, swabbing of PWID for non-invasive GAS, referral of isolates to RVPBRU for typing, emphasising safe and hygienic injection practices when communicating with PWIDs and ensuring their easy access to needle and syringe programmes (NSP).

In towns with multiple cases, local OCTs reviewed the policies and practices of affected NSP and homeless hostels around injecting, infection control and environmental cleaning, comparing them against national guidance [1,2] and published evidence [6]. Teaching sessions were organised for frontline homeless service providers to raise awareness of GAS infection and infection control measures. Targeted communications to raise awareness of early symptoms and to encourage prompt healthcare attendance in the event of skin problems at injection sites were disseminated via general practitioners, local authorities, pharmacies and included in the equipment packs of one NSP.

## Discussion

Large outbreaks of GAS type *emm*66 have not previously been described. In this outbreak although the cases occurred disproportionately among PWID, transmission appears unrelated to drug usage. Illness occurred over an 11-month period, cases were representative of the wider UK PWID population in terms of sex, age and hepatitis C prevalence [3] and we identified no notable changes in drug using practice in the period before illness. The age and sex distribution of cases, low mortality (only one case died) and predominance of abscesses and injection site infections were also broadly consistent with the pattern seen among iGAS cases in PWID in England in the early 2000s [7,8].

We have identified potential transmission clusters within three of the affected towns but only limited epidemiological links between cases in different towns. Travel along the major transport routes connecting the towns remains a plausible hypothesis for the disease propagation observed. Whole genome sequencing (WGS) of outbreak and historic *emm*66 isolates held by RVPBRU may have potential to identify links between cases, improve the specificity of our case definition and establish whether the outbreak strain has any genes suggestive of increased virulence. However, the role of WGS in an outbreak of such a rare GAS type, with limited availability of historical isolates for comparison, is the subject of ongoing discussion.

Difficulties in interviewing the affected population, especially for cases identified retrospectively, and accurately establishing networks of contacts pose challenges for investigation and control. Close coordination between local HPTs and frontline drug/homeless outreach services has been essential for accessing the affected population and implementing control measures to date.

The proportion of iGAS infections reported to RVPBRU attributed to PWID (other risk factors are not recorded) has increased annually since 2013 from 0.2% to 1.7% in 2016. A previous rise, dominated by GAS type *emm*83, was recorded in the early 2000s, with PWID accounting for 20% of all iGAS cases in England and Wales at its peak in 2003 [8,9]. The requirement for iGAS isolate submission has not changed during 2013–16 and it is unclear whether the increase represents a true change in disease burden among PWID, increased awareness of injection site infections and/or access to healthcare, or if it is an artefact of increased PWID reporting on referral forms. Prior to the increase in GAS type *emm*66 in 2016, the most common type seen among PWID with iGAS was *emm*94, with six cases in the entire period 2010–15 and two cases in 2016. GAS type *emm*66 is very uncommon in high income settings such as Europe and North America [10-12]. It comprised 1.3% of iGAS isolates submitted to RVPBRU for typing in 2016; an increase from 0.16% of the isolates during 2010–15. There is very limited mention of type *emm*66 in the literature beyond a single case in Hungary in 2004–05 [13] and sequencing of an isolate from within a cluster of 13 cases in France in 2009–13 [14]. The rapid increase in the number of type *emm*66 iGAS cases detected in England and Wales in 2016 and the high proportion of PWID among cases leave us confident that this is a genuine outbreak. So far, despite the wide geography, the outbreak has been limited to this vulnerable marginalised group and *emm*66 may now be the dominant circulating type within that population. Unfortunately, there are no data on background carriage rates of *emm* types within different groups.

There is no indication of a drug-related vehicle of infection and we can conclude that there have been no common risk factors identified to date other than those listed in the case definition. These are all associated with increased vulnerability to iGAS and may result in protracted incidence as previously described in a Canadian GAS type *emm*59 outbreak [15]. We must therefore maintain increased awareness of the early signs of GAS infection among the affected population and those that serve them. As our investigations continue, we expect lessons from this outbreak to emerge around transmission routes and effectiveness of control measures that will have relevance to other countries facing GAS outbreaks in vulnerable, under-served populations.
